# Mitochondrial Structure and Polarity in Dendrites and the Axon Initial Segment Are Regulated by Homeostatic Plasticity and Dysregulated in Fragile X Syndrome

**DOI:** 10.3389/fcell.2021.702020

**Published:** 2021-07-19

**Authors:** Pernille Bülow, Peter A. Wenner, Victor Faundez, Gary J. Bassell

**Affiliations:** ^1^Department of Cell Biology, Emory University School of Medicine, Atlanta, GA, United States; ^2^Department of Physiology, Emory University School of Medicine, Atlanta, GA, United States

**Keywords:** *Fmr1*, FMRP, mitochondria, homeostatic plasticity, compartment-specific, dendrite, axon initial segment

## Abstract

Mitochondrial dysfunction has long been overlooked in neurodevelopmental disorders, but recent studies have provided new links to genetic forms of autism, including Rett syndrome and fragile X syndrome (FXS). Mitochondria show plasticity in morphology and function in response to neuronal activity, and previous research has reported impairments in mitochondrial morphology and function in disease. We and others have previously reported abnormalities in distinct types of homeostatic plasticity in FXS. It remains unknown if or how activity deprivation triggering homeostatic plasticity affects mitochondria in axons and/or dendrites and whether impairments occur in neurodevelopmental disorders. Here, we test the hypothesis that mitochondria are structurally and functionally modified in a compartment-specific manner during homeostatic plasticity using a model of activity deprivation in cortical neurons from wild-type mice and that this plasticity-induced regulation is altered in *Fmr1*-knockout (KO) neurons. We uncovered dendrite-specific regulation of the mitochondrial surface area, whereas axon initial segment (AIS) mitochondria show changes in polarity; both responses are lost in the *Fmr1* KO. Taken together, our results demonstrate impairments in mitochondrial plasticity in FXS, which has not previously been reported. These results suggest that mitochondrial dysregulation in FXS could contribute to abnormal neuronal plasticity, with broader implications to other neurodevelopmental disorders and therapeutic strategies.

## Introduction

Mitochondria are essential for regulating cellular metabolism and maintaining neuronal health, and abnormal mitochondrial function is associated with altered neuronal development and disease (Valenti et al., 2014). More recently, studies have demonstrated impairments in mitochondrial structure and function in various neurodevelopmental mouse models of autism, including fragile X syndrome (FXS). Mitochondria are fragmented and depolarized in neurites of the FXS mouse model, the *Fmr1* knockout (KO), and the *Drosophila*, *dFmr1* (Weisz et al., 2018; Shen et al., 2019). Thus, restoring mitochondria function is an exciting path for future therapeutic strategies. However, we still do not know how these impairments in mitochondrial structure/function contribute to symptoms in disease.

Fragile X syndrome is characterized by intellectual disability, sensory hypersensitivities, and seizures. Moreover, the mutation causing FXS is one of the leading monogenic causes of autism. One of the core phenotypes in FXS animal models is abnormal synaptic, structural, and functional plasticity (Huber et al., 2002; Meredith et al., 2007; Soden and Chen, 2010; Bulow et al., 2019). We recently reported cell-type-specific abnormalities in homeostatic intrinsic plasticity (HIP) in *Fmr1*-KO neurons, where some cells displayed exaggerated HIP while HIP was absent in others (Bulow et al., 2019). HIP is critical for maintaining neuronal activity levels by regulating the intrinsic membrane excitability of neuronal membranes. Another form of homeostatic plasticity is called synaptic scaling, which regulates neuronal activity by altering synaptic function. One group found that synaptic scaling is absent in hippocampal *Fmr1*-KO neurons (Soden and Chen, 2010). While HIP can regulate intrinsic excitability by altering ion channel expression/function at the axon initial segment (AIS), synaptic scaling regulates the expression of neurotransmitter receptors in dendrites (Turrigiano, 2012). Thus, *Fmr1*-KO neurons display compartment-specific abnormalities in distinct forms of homeostatic plasticity.

Interestingly, a number of studies have demonstrated that mitochondria may support neuronal plasticity in dendrites and axons (Rangaraju et al., 2019; Kuzniewska et al., 2020). Moreover, studies have reported morphological changes in mitochondria in response to neuronal plasticity (Li et al., 2004; Chang et al., 2006). Specifically, dendrite-residing mitochondria become larger after activity silencing but become fragmented following increases in activity (Li et al., 2004; Chang and Reynolds, 2006). Currently, it remains unknown how mitochondrial shape/function is regulated at the molecular level during activity. We know that the synthesis of nuclear encoded mitochondrial protein is upregulated during a chemical LTP paradigm in synaptoneurosomes (Kuzniewska et al., 2020), and the incorporation of these proteins into the mitochondrion appears necessary for synaptic plasticity. Fragile X mental retardation protein (FMRP), the protein lost in FXS, is best known as a translational repressor, and FMRP targets ∼1.4% of all mitochondrial annotated mRNAs (Suhl et al., 2014; Richter et al., 2015; Rath et al., 2021). Upon changes in neuronal activity, FMRP derepresses its mRNA targets, allowing for translation and upregulation (Muddashetty et al., 2011). FMRP may regulate the translation of mitochondrial proteins in an activity-dependent manner, or alternatively, the effect on mitochondria may be downstream of the effect of FMRP on protein synthesis. Here, we test the hypothesis that loss of FMRP impairs mitochondrial plasticity. To date, it remains completely unknown if and how defects in mitochondrial structure/function in *Fmr1*-KO neurons impair neuronal plasticity. Specifically, we address how the *Fmr1* mutation causing FXS affects mitochondrial plasticity during a homeostatic plasticity paradigm.

Homeostatic intrinsic plasticity and other forms of homeostatic plasticity are expressed in compartment-specific manners and are both protein synthesis dependent (Sutton et al., 2006; Muraro et al., 2008). HIP is often associated with changes in the AIS (Grubb and Burrone, 2010; Kuba et al., 2010), while synaptic scaling, a type of homeostatic plasticity which regulates synaptic strength, is notable on postsynaptic spines in dendrites (Turrigiano et al., 1998; Sutton et al., 2006). Thus, the same activity perturbation (e.g., activity deprivation) can trigger multiple types of homeostatic mechanisms that express in different neuronal compartments. Previously, most work has focused on how mitochondria are regulated by activity in dendrites (Li et al., 2004; Chang et al., 2006; Kuzniewska et al., 2020). However, the axon, particularly the AIS, is essential for regulating neuronal excitability and understanding how the mitochondrion contributes to plasticity at the AIS and may be especially important for dysregulated excitability in many models of FXS and ASD (Contractor et al., 2015). Therefore, we tested the hypothesis that activity perturbation triggers compartmentalized changes in the mitochondrion structure and function and that this mitochondrial plasticity is altered in *Fmr1*-KO neurons.

## Methods

### Mice

*FMR1*^*HET*^ female mice (backcrossed on C57BL6 background, B6.129P2-*Fmr1*tm1Cgr/J; Stock No: 003025) were crossed with WT C57BL6 males (Jackson Laboratory) to generate litters of pups with mixed genotypes [*Fmr1*^–/y^, *Fmr1*^*HET*^, or wild type (WT)]. Thus, for all experiments, *Fmr1*-KO male pups were compared to their WT littermate control. We performed PCR to identify genotypes on postnatal days 0–1 (P0–P1) as described previously. The mice were housed in a 12-h light/dark cycle, and the animal protocol was approved by the Institutional Animal Care and Use Committees at Emory University.

### Primary Cortical Neuronal Cultures

Cerebral cortices were dissected and cultured from genotyped WT and *Fmr1*^–/y^ pups on P0–P1. The cortices were enzymatically dissociated using trypsin (Thermo Fisher Scientific, cat no.: 15050-065; 10 min), mechanically dissociated in Minimum Essential Media (MEM; Fisher, cat no.: 10-010-CV) supplemented with 10% fetal bovine serum (FBS; HyClone, cat no.: SH30070.03), and stained to assess viability using Trypan Blue (Sigma, cat no.: T10282). The neurons were plated in one of two ways: For immunocytochemical experiments, the neurons were plated on coverslips (Matsunami Inc., cat no.: C022001; 22 mm) coated with FBS, poly-*l*-lysine and laminin. A total of 35,000 neurons were plated as a “spot” on the center of the coverslip to create a small, high-density network. For live-cell imaging, 100K neurons were plated on 35-mm glass-bottom MatTek Petri dishes (MatTek Corp., cat no.: P35G-1.5-14-C) coated with FBS, poly-lysine (Sigma, cat no.: P5899), and laminin (Sigma, cat no.: L2020). The neurons were cultured in standard growth medium [glial conditioned neurobasal (Fisher, cat no.: A3582901) supplemented with GlutaMAX (Gibco, cat no.: 35050061) and B27 (Gibco, cat no.: A3653401)], and half of the media was exchanged two to three times a week until experimental treatments began. No antibiotics or antimycotics were used. The cultures were maintained in an incubator regulated at 37°C, 5% CO_2_, and 95% relative humidity. Culturing protocol is similar to that of Bulow et al. (2019). All experiments were performed with neuronal cultures at 12 days *in vitro* (DIV).

### Pharmacology

Drugs were used in the following concentrations (in μM): tetrodotoxin (TTX), 1 (Tocris, cat no.: 1069), which was dissolved in molecular-grade H_2_O; and D-(-)-2-amino-5-phosphonopentanoic acid (APV), 100 (Tocris, cat no.: 0106), which was dissolved into 0.1 M NaOH. Drugs were co-applied to fresh standard growth medium and added to the cultures by a complete media change on DIV 10 and lasted for 48 h. The treatment drugs were refreshed after 24 h. Control cultures had a simultaneous complete media change but without drugs. Cultures were randomly assigned to each treatment group (control or TTX/APV). All experiments occurred on DIV 12.

### Immunocytochemistry of Mitochondrial Morphology and AIS Colocalization

The coverslips were washed once at 37°C in phosphate-buffered saline (PBS) and fixed with 4% paraformaldehyde (mixed with PBS) for 15 min at 37°C. The cultures were washed three times, permeabilized with 0.2% Triton in 1 × PBS for 10 min at room temperature (RT), washed with 20%/0.15% Tris (Corning, cat no.: 46-030-CM)–glycine (Sigma, cat no.: 8001) in 1 × PBS for 5 min, and blocked for 1 h in a 5% bovine serum albumin (Roche Diagnostics, cat no.: 03116964001) solution. The coverslips were incubated with the primary antibodies overnight at 4°C, washed three times, incubated with secondary antibodies for 1 h at RT, and mounted. All imaging occurred within the first week after mounting.

The primary antibodies used included Hsp60 (D6F1) (Cell Signaling, cat no.: 12165S), ankyrin G (NeuroMabs, cat no.: 75-146), MAP2 (Synaptic Systems, cat no.: 188-004), and neurofascin (Cell Signaling, cat no.: 15034).

The secondary antibodies used included Alexa Fluor 488 (Life Technologies, cat no.: A21206), IgG Cy3 (Jackson ImmunoResearch, cat no.: 715-165-150), and Cy5 AffiniPure (Jackson ImmunoResearch, cat no.: 715-175-150).

All antibodies were used at 1:500 dilution.

### Imaging, Image Processing, and Imaging Analysis of Mitochondrial Morphology and AIS Colocalization

Imaging was performed on a Nikon Eclipse Ti-E inverted microscope with Z-stacks measuring 0.2-μm steps (around 10 total steps) with a × 60 magnification lens. The images were deconvoluted with the AutoQuant X3.1 software and analyzed in ImageJ/FIJI (version 2.0.0-rc-69/1.52n) using the plug-in “Mito-Image J” (Wiemerslage and Lee, 2016). Each neuron had the following compartments outlined: soma, dendrite (15 μm away from the soma, 15 μm in length, three to four dendrites analyzed per neuron), and AIS (defined as described by Evans et al. (2013). For the mitochondrial stain, we picked the three stacks in best focus and converted these into a max-intensity projection. The mitochondrial channel was thresholded automatically using the thresholding program “ISODATA.” All mitochondria detected by the program were confirmed by eye.

### TMRM Live-Cell Imaging

TMRM was acquired from Thermo Fisher Scientific (cat no.: T668) and diluted to 10 nm in fresh cell culture medium. The TMRM medium was applied to the cell cultures for 20 min at 37°C. The cultures were washed once in warmed imaging buffer (137 mM NaCl, 0.56 mM MgCl, 4.7 mM KCl, 1.28 mM CaCl, 1.28 mM glucose, 1 mM NaPO_4_, and 10 mM Hepes). OxyFluor (Millipore, cat no.: SAE0059) was added on the day of imaging. The live-cell imaging was performed on a Nikon TE2000 inverted microscope with a × 60 magnification lens at 37°C, and the imaging session lasted 30 min at the maximum.

To identify the AIS, we first incubated the cell cultures with a primary antibody against the extracellular domain of neurofascin (Cell Signaling, cat no.: 15034, 1:500) for 5 min at 37°C. We washed the cells twice with a fresh culture medium, added a secondary antibody (Alexa Fluor 488, Life Technologies, cat no.: A21206) for 1 min at RT in the dark, then washed them once with a fresh cell culture medium, added the TMRM medium (10 nm), and followed the protocol described for neurites.

The TTX/APV-treated cultures had TTX/APV added to the incubation medium at all steps. FCCP (0.5 μM) (carbonyl cyanide-4-(trifluoromethoxy)phenylhydrazone), Sigma, cat no.: C2920) was added 5 min into the recordings.

### Image Analysis of TMRM Intensity

The images were analyzed in FIJI (version 2.0.0-rc-69/1.52n). The dendritic region of interest was defined as 15 μm away from the soma and 15 μm in length. Two to four dendrites were analyzed per cell. The AIS was outlined based on the co-labeled neurofascin stain. The area and mean intensity of the ROIs were quantified in FIJI.

### Statistics and Biological Replicates

**FIGURE 1:**
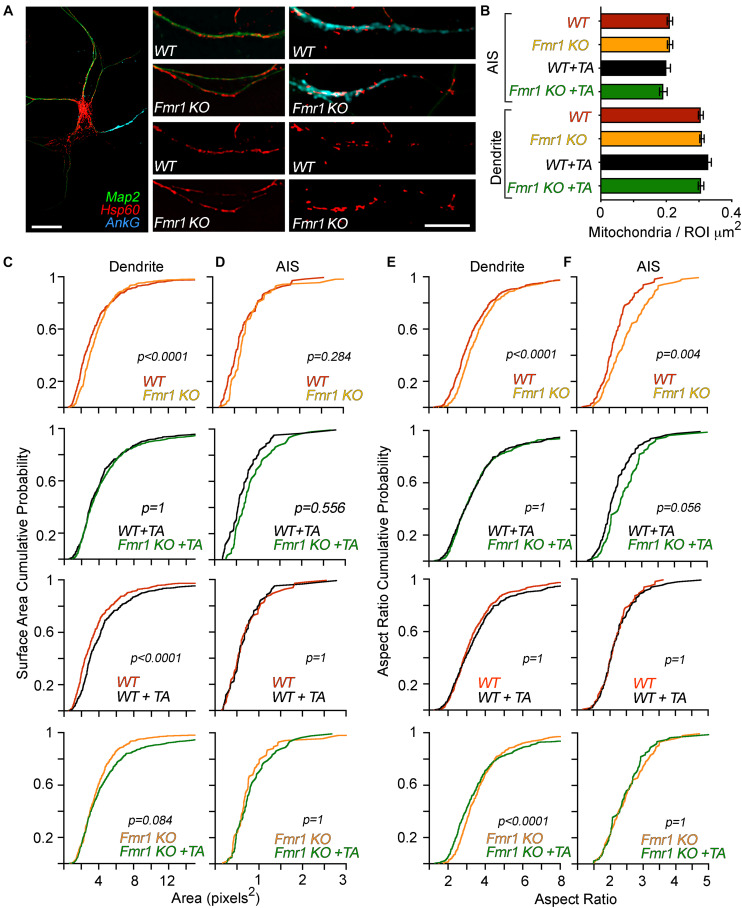
Genotype-specific, compartment-specific, and treatment-specific alterations in mitochondrial morphology. Layout: the top row is composed of representative images and quantification of the number of mitochondria in dendrites and AIS, respectively. Underneath, four rows comprise data on mitochondrial morphology. Each vertical row has the following presentation of data comparisons: WT control vs. *Fmr1*-KO control, WT + TTX/APV vs. *Fmr1*-KO + TTX/APV, WT control vs. WT + TTX/APV, and *Fmr1*-KO control vs. *Fmr1*-KO + TTX/APV. **(A)** Representative images of immunofluorescence co-labeling of Hsp60, Map2, and Ank G. Scale bars = 20 μm. From left: full neuron, dendrite, AIS. **(B)** Quantification of mitochondrial content in dendrites and AIS. There were no differences in content of mitochondria between any conditions. Error bars represent the standard error of the mean. **(C)** Quantification and comparison of dendritic mitochondrial surface area before and after TTX/APV treatment. At baseline, mitochondria display a significantly larger surface area in *Fmr1* KO compared to WT. TTX/APV increases mitochondrial surface area in WT dendrites. *Fmr1*-KO mitochondria display a non-significant trend toward an increase of surface area following TTX/APV. **(D)** Quantification and comparison of AIS mitochondrial surface area before and after TTX/APV treatment. There are no differences in surface area between any conditions before or after TTX/APV. **(E)** Quantification and comparison of dendritic mitochondrial aspect ratio between genotypes before and after TTX/APV treatment. At baseline, the mitochondrial aspect ratio is increased in *Fmr1*-KO neurons, but this difference is abolished after TTX/APV. Interestingly, *Fmr1*-KO mitochondria display a small but significant increase in the aspect ratio after TTX/APV, while no changes occur in the WT. **(F)** Quantification and comparison of AIS-residing mitochondrial aspect ratio before and after TTX/APV treatment. At baseline, mitochondria display increased aspect ratio in *Fmr1*-KO AIS compared to WT, but this difference is lost after TTX/APV treatment. All figures represent data from *N* = 3 biological replicates from independent littermate culture sets. Each analyzed dendrite and AIS represents an individual data point. *N* of analyzed dendrites: WT *n* = 400, *Fmr1* KO *n* = 427, WT + TA *n* = 366, *Fmr1*-KO + TA *n* = 377. *N* of AIS: WT *n* = 90, *Fmr1*-KO *n* = 80, WT + TA *n* = 74, *Fmr1*-KO + TA *n* = 69. TA = TTX/APV.

**FIGURE 2:**
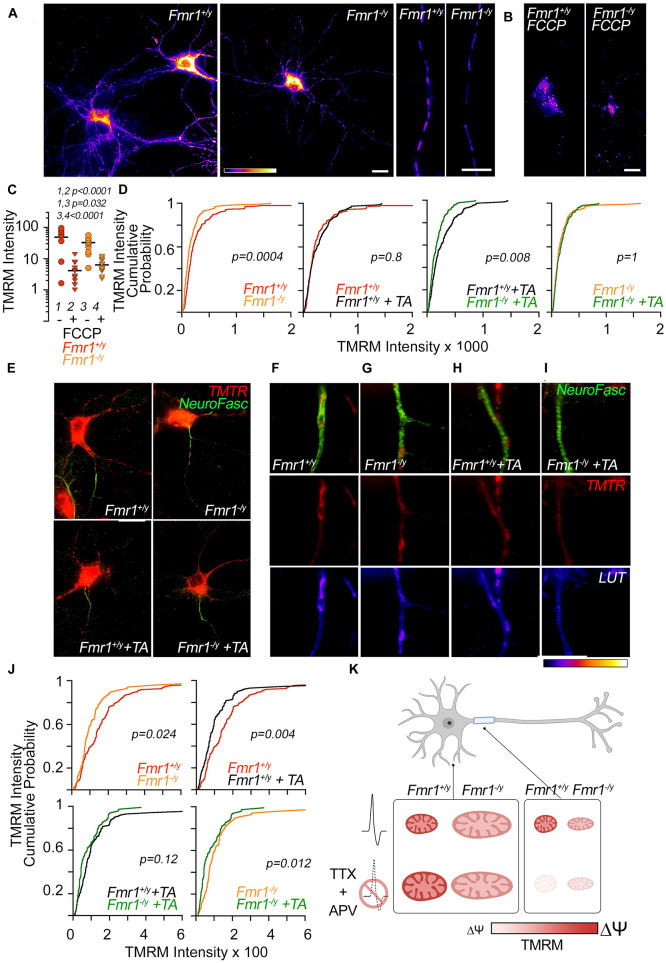
Compartmentalized membrane potential modifications in WT and *Fmr1*-KO mitochondria before and after activity deprivation. Layout: the top half of the figure comprises data on mitochondrial membrane potential in the dendrite. The bottom half of the figure comprises data on mitochondrial membrane potential in the AIS. The bottom right contains a summary diagram of the described results in Figure 1. **(A)** Representative images of TMRM-labeled neurons. Scale bars = 20 μm. From left: full neurons either in control conditions or after FCCP (0.5 μM) treatment, representative dendritic segments. **(B,C)** FCCP treatment caused significantly reduced TMRM signals in dendritic mitochondria of both WT (1, 2) and *Fmr1* KO (3, 4); + = FCCP, - = no FCCP. **(D)** TMRM intensity is significantly reduced in dendrites of *Fmr1* KO compared to WT at baseline and after TTX/APV treatment. TMRM signal in dendrites was not affected by TTX/APV treatment in either genotype. **(E)** Representative images of co-labeled neurons with TMRM and AIS-specific marker neurofascin from all conditions. **(F–I)** Top: Representative images of neurofascin-positive neurites colocalizing with TMRM-labeled mitochondria. Middle: same neurite segment as in the top row but with the neurofascin stain removed to isolate the colocalizing TMRM-labeled mitochondria. Bottom: same images as above but pseudo-colored according to TMRM-labeled fluorescence intensity. Row **(F)** = WT, **(G)** = *Fmr1* KO, **(H)** = WT + TTX/APV, **(I)** = *Fmr1* KO + TTX/APV. **(J)** At baseline, AIS-residing *Fmr1*-KO mitochondria display significantly reduced TMRM intensity. Following TTX/APV, mitochondria in the AIS significantly reduce their TMRM signal in both genotypes. However, the TTX/APV-induced reduction in TMRM signal is of greater magnitude in the WT compared to *Fmr1* KO. *N* of analyzed dendrites: WT *n* = 210, *Fmr1* KO *n* = 175, WT treated with TTX/APV *n* = 179, *Fmr1* KO treated with TTX/APV *n* = 187. *N* of AIS: WT *n* = 75, *Fmr1* KO *n* = 62, WT + TA *n* = 67, *Fmr1* KO + TA *n* = 59. **(K)** Summary model: compartment-specific regulation of mitochondrial morphology and polarization by FMRP and activity deprivation. After TTX/APV, WT mitochondria are enlarged in the dendrites and depolarized in the AIS. At baseline, *Fmr1*-KO neurons present with enlarged and/or elongated mitochondria in the dendrite and AIS, respectively, and mitochondria in both compartments display depolarized membrane potential. After TTX/APV, *Fmr1*-KO mitochondria do not show changes in their size and only a slight further reduction in polarization. TA = TTX/APV.

The morphology and TMRM intensity data sets were evaluated using the Kolmogorov–Smirnov statistical test followed by Bonferroni corrections (alpha = 0.05). All *p*-values are listed in the figures. Experiments for mitochondrial morphology (Figure 1) were repeated in three independent experiments. Approximately 40 neurons were analyzed for each condition per experiment. Experiments for mitochondrial membrane polarity (Figure 2) were repeated in three independent experiments for mitochondria in the dendrites. Approximately 20 neurons were analyzed for each condition per experiment. For the AIS, experiments were repeated in four independent experiments comparing WT and *Fmr1*-KO neurons. WT cultures were repeated an extra time without a *Fmr1*-KO comparison, leading to a total of five independent biological replicates for the WT, and four independent biological replicates for the *Fmr1* KO. Approximately 12 neurons were analyzed for each condition per experiment. All error bars represent standard errors of the mean. All data analysis was performed in FIJI (version 2.0.0-rc-69/1.52n), and all statistics were performed using the Kolmogorov–Smirnov statistical test software on http://www.physics.csbsju.edu/stats/KS-test.n.plot_form.html.

## Results and Discussion

We previously triggered homeostatic plasticity in DIV 12 WT and *Fmr1*-KO cortical primary neurons by co-applying TTX and APV for 48 h (Bulow et al., 2019). This treatment is often used to trigger protein synthesis-dependent forms of homeostatic plasticity (Sutton et al., 2006; Soden and Chen, 2010). Here, we utilize the same pharmacological approach to trigger homeostatic plasticity in *Fmr1*-KO and WT cortical neurons to uncover how mitochondria are affected by activity chronic deprivation and the loss of *FMR1*. After 48 h of treatment, we performed immunocytochemistry to visualize the mitochondrial morphology in dendrites and the AIS using a primary antibody against the mitochondrial chaperone protein Hsp60 (Figure 1). We previously reported that Hsp60 protein levels were unaffected by genotype or treatment determined by mass spectrometry (Bulow et al., 2021). In another set of experiments, we tested compartmental changes in mitochondrial function by performing live-cell imaging to visualize the mitochondrial membrane potential in dendrites or the AIS (Figure 2). The AIS was identified by using antibodies against the AIS-enriched membrane protein neurofascin, which is well reported to overlap with ankyrin G staining in neurons (Evans et al., 2015). Using immunocytochemistry, we validated that neurofascin expression overlaps with ankyrin G (data not shown). In general, we quantified larger and more numerous mitochondria in the dendrite compared to the AIS (Figure 1B), consistent with previous literature describing differences between mitochondria in dendrites and axons (Chang et al., 2006). In accordance with our hypothesis, we observed compartment-specific changes in the mitochondrial morphology (Figure 1) and membrane potential (also referred to as the “polarity”) in WT neurons (Figure 2). Specifically, after TTX/APV, WT neurons displayed a unique increase in the surface area of dendrite-residing mitochondria but not in mitochondria within the AIS (Figures 1C,D). Conversely, the mitochondrial polarity was distinctly reduced in AIS-residing mitochondria, but not in mitochondria within the dendrite (Figures 2D,J). Thus, 48-h activity deprivation led to compartment-specific changes in the morphology and polarity of mitochondria in WT neurons. We hypothesize that these compartment-specific changes support the expression of distinct types of homeostatic plasticity. For example, we speculate that the morphological changes in dendrite-residing mitochondria suggest changes in functional activity of the mitochondria that could support synaptic plasticity. Alternatively, synaptic scaling may trigger downstream mechanisms that lead to enlargement of the mitochondria, perhaps to maintain the increased synaptic size that occurs during synaptic scaling. Both of these speculations may account for the mitochondrial contributions to synaptic/structural plasticity observed in recent papers (Rangaraju et al., 2019; Kuzniewska et al., 2020). In contrast, we speculate that reduced polarity of AIS-residing mitochondria after TTX/APV affects mitochondrial buffering of intracellular Ca^++^. A reduced membrane potential can suggest uncoupling of the mitochondrial electron transport chain (ETC) which could impair mitochondrial Ca^++^ buffering or, alternatively, increased Ca^++^ entry into the mitochondria. Ca^++^ signaling is critical for the expression of homeostatic plasticity, including HIP (Lee et al., 2015), and reduced or increased intracellular Ca^++^ could influence pathways regulated by neuronal activity. Irrespective of the directionality of the changes we observe in mitochondria, our study measures an intriguing correlation between compartmentalized changes in mitochondrial morphology/function and the expression of distinct types of homeostatic plasticity (Bulow et al., 2019).

Consistent with previous reports, we quantified alterations in both the morphology and membrane potential of *Fmr1*-KO neurons at baseline. In dendrites, mitochondria had a larger surface area, while mitochondria were longer in the AIS of *Fmr1*-KO neurons compared to WT (Figures 1C,D, top panels). This increase in mitochondrial size contrasts with previous studies reporting smaller and rounder mitochondria in animal models of FXS (Weisz et al., 2018; Shen et al., 2019), although one study using human neurons found no differences in mitochondrial morphology between patient cells and controls (Nobile et al., 2020). There are several possibilities that could account for the discrepancy in our results compared to others. (1) The increased size may represent mitochondrial “swelling,” which is a phenotype typically associated with dysfunctional mitochondria. (2) The mitochondria observed may represent “clumps” of fragmented mitochondria. While this is possible, our technical approach to measure mitochondrial morphology is similar to previous studies (Shen et al., 2019), thereby supporting the soundness of our findings. (3) Previous studies have used *dFmr1* flies (Weisz et al., 2018) or *Fmr1*-KO adult hippocampal stem cells (Shen et al., 2019) to assess mitochondrial morphology. Our study uses DIV 12 cortical primary neurons which likely represent a distinct developmental time point of brain development. Mitochondrial shape and function change during brain development (Khacho et al., 2016), and we hypothesize that defects in mitochondrial morphology in FXS are developmentally regulated. In contrast with the enlarged (rather than the typically fragmented) size of *Fmr1*-KO mitochondria, we did measure reductions in the membrane polarity of both dendrite- and AIS-residing mitochondria (Figures 2D,J), which is a result consistent with previous reports (Shen et al., 2019; Licznerski et al., 2020).

A main finding of our study is that *Fmr1*-KO neurons have altered mitochondrial plasticity in compartment-specific manners compared to WT. First, we observed that in contrast to WT mitochondria, *Fmr1*-KO dendritic mitochondria displayed no further enlargement after TTX/APV treatment (Figure 1C, bottom panel). Second, while *Fmr1*-KO AIS-residing mitochondria did display a small decrease in membrane potential after TTX/APV, this difference was a fraction of the observed reduction in the WT (Figure 2J, compare the bottom-right panel to the top-right panel). In fact, after TTX/APV treatment, WT and KO mitochondria in the AIS no longer differed in polarity (Figure 2J, bottom-left panel). In sum, *Fmr1*-KO mitochondria display altered sensitivity to neuronal activity in both dendrites and AIS. We hypothesize that FMRP regulates mitochondrial morphology and polarity in an activity-dependent manner and that loss of FMRP impairs this process. We further speculate that the loss of mitochondrial plasticity may contribute to or be a result of abnormal homeostatic plasticity expression. It is possible that mitochondrial plasticity is impaired in *Fmr1*-KO neurons because the mitochondria are already phenocopying WT mitochondria during plasticity. This idea aligns with previous work suggesting that FMRP primarily is an activity-sensitive molecule which plays a critical role in mediating molecular pathways during plasticity (Osterweil et al., 2010; Niere et al., 2012). Indeed, we recently reported that FMRP restricts proteome changes during homeostatic plasticity (Bulow et al., 2021). Loss of FMRP led to a dramatic increase in proteomic changes following TTX/APV treatment. Surprisingly, we did not observe major differences in the neuronal proteome between *Fmr1*-KO and WT cultures at baseline in our proteomic analysis, despite the fact that we observed differences in morphology and polarity of KO mitochondria at baseline (see the summary diagram in Figure 2K). We speculate that loss of FMRP may not impair mitochondrial protein expression sufficiently to reach statistical significance at baseline in our rigorous proteomic study. However, following activity deprivation, proteomic differences are either triggered or exaggerated such that the proteome changes consistently achieve statistical significance. Further experiments are needed to test if and how the mitochondrial protein composition may change without further increasing the surface area or dramatically decreasing polarity. One possibility is that while morphology and polarity are unaffected by TTX/APV treatment, other changes occur in signaling. For example, cellular stress is known to trigger the release of reactive oxygen species (ROS), which can be detrimental for neuronal health. Studies should test if ROS is increased in *Fmr1*-KO neurons following TTX/APV. We hypothesize that abnormal mitochondrial plasticity impairs the expression of HIP in *Fmr1*-KO neurons previously reported by our lab (Bulow et al., 2019).

In summary, our work confirms our hypothesis that mitochondrial structure and function are regulated in a compartment-specific manner during homeostatic plasticity in WT neurons but impaired in *Fmr1*-KO mitochondria. Mitochondrial abnormalities at baseline may be directly or indirectly caused by loss of FMRP expression. Further work is necessary to understand the underlying molecular mechanism of how FMRP regulates mitochondrial plasticity, when mitochondrial defects arise in FXS, and lastly how restoring mitochondrial plasticity can rescue neuronal plasticity.

## Data Availability Statement

The raw data supporting the conclusions of this article will be made available by the authors, without undue reservation.

## Ethics Statement

The animal study was reviewed and approved by the IACUC, Emory University.

## Author Contributions

PB, GB, PW, and VF conceived of the experimental design. PB completed all the experiments. PB, VF, and GB prepared the manuscript and figures. All authors contributed to the article and approved the submitted version.

## Conflict of Interest

The authors declare that the research was conducted in the absence of any commercial or financial relationships that could be construed as a potential conflict of interest.

## Abbreviations

AIS: axon initial segment
APV: D-(–)-2-amino-5-phosphonopentanoic acid
ATP: adenosine triphosphate
DIV: days *in vitro*
FMRP: fragile X mental retardation protein
FXS: fragile X syndrome
HIP: homeostatic intrinsic plasticity
KO: knockout
LTP: long-term potentiation
ROS: reactive oxygen species
TTX: tetrodotoxin
WT: wild type.
